# Professional View of the Death of Commodore M. F. Maury

**Published:** 1873-02

**Authors:** B. S. Herndon

**Affiliations:** Savannah, Georgia


### PROFESSIONAL VIEW OF THE DEATH OF COMMODORE M. F. MAURY.

BY B. S. HERNDON, M.D., SAVANNAH, GEORGIA.

It may interest your readers to know some of the particulars of the illness of the late Commodore Maury—both because of the personal character of the deceased, who was so widely known, and so generally esteemed throughout the country and the world, and because of the interest of the case considered medically.

After a long and fatiguing travel from St. Louis, in the month of September, he was seized with acute gastric disease, manifested by vomiting and pain. The Commodore had for many years been a sufferer from gout, and at first his medical attendant considered that he was probably laboring under an attack of his old enemy; but the persistence of his trouble, together with emesis of blood and bloody passages, induced Dr. Madison to pronounce his malady chronic ulcer of the stomach. (*Vide* Nemeyer, vol. i.) He had never at any time a particle of fever. He complained of constant dull, and sometimes acute, pain in the epigastrium, much aggravated by taking food of any kind, except stale bread and milk, upon which he lived during the greater part of his illness.

Week after week passed, with alternations of improvement and relapse, his strength all the time wasting. He experienced comfort from hot applications, and preferred warm drinks. Much drug treatment was not attempted. He took carbolic acid in glycerine, as a topical application, and some trial was made of the usual course of remedies; but he wearied of them, and received no benefit. Death resulted from inanition. His faculties were unclouded to the last. He expressed the utmost resignation to the Divine will, and bore testimony to the exceeding comfort of an humble faith in Christ. Surrounded by his loved ones, he gave them his blessing, and on the 1st February, at the age of sixty-seven, gently passed from earth, at peace with God and in perfect charity with all mankind. Looking from his window, a short time before his death, upon beautiful Venus, he said to his wife: "My dear, perhaps my spirit may go to that star." It was suggestive of the Star of Bethlehem.

